# Identification of Protein Biomarkers for Cervical Cancer Using Human Cervicovaginal Fluid

**DOI:** 10.1371/journal.pone.0106488

**Published:** 2014-09-12

**Authors:** Geert A. A. Van Raemdonck, Wiebren A. A. Tjalma, Edmond P. Coen, Christophe E. Depuydt, Xaveer W. M. Van Ostade

**Affiliations:** 1 Laboratory for Protein Science, Proteomics and Epigenetic Signaling (PPES) and Centre for Proteomics and Mass spectrometry (CeProMa), University of Antwerp, Wilrijk, Belgium; 2 Department of Gynaecology and Gynaecologic Oncology, University Hospital Antwerp, Edegem, Belgium; 3 Department of Molecular Diagnostics, Algemeen Medisch Laboratorium bvba, Sonic Healthcare Benelux, Antwerpen, Belgium; The University of Queensland, Australia

## Abstract

**Objectives:**

Cervicovaginal fluid (CVF) can be considered as a potential source of biomarkers for diseases of the lower female reproductive tract. The fluid can easily be collected, thereby offering new opportunities such as the development of self tests. Our objective was to identify a CVF protein biomarker for cervical cancer or its precancerous state.

**Methods:**

A differential proteomics study was set up using CVF samples from healthy and precancerous women. Label-free spectral counting was applied to quantify protein abundances.

**Results:**

The proteome analysis revealed 16 candidate biomarkers of which *alpha-actinin-4* (p = 0.001) and *pyruvate kinase isozyme M1/M2* (p = 0.014) were most promising. Verification of *alpha-actinin-4* by ELISA (n = 28) showed that this candidate biomarker discriminated between samples from healthy and both low-risk and high-risk HPV-infected women (p = 0.009). Additional analysis of longitudinal samples (n = 29) showed that *alpha-actinin-4* levels correlated with virus persistence and clearing, with a discrimination of approximately 18 pg/ml.

**Conclusions:**

Our results show that CVF is an excellent source of protein biomarkers for detection of lower female genital tract pathologies and that *alpha-actinin-4* derived from CVF is a promising candidate biomarker for the precancerous state of cervical cancer. Further studies regarding sensitivity and specificity of this biomarker will demonstrate its utility for improving current screening programs and/or its use for a cervical cancer self-diagnosis test.

## Introduction

The human papillomavirus (HPV) is responsible for virtually all cervical cancers [1]. Although more than 150 variants of this virus exist, only certain genotypes, such as HPV 16, 18, 33, 45 and 58 are known as high-risk types (HR-HPV) [2]. Low-risk HPV types (LR-HPV), mainly HPV 6 and 11, seldom cause genital tumors; however, they do cause condylomata acuminata (anogenital warts) [3]. Persistent HPV oncoprotein expression (E6/E7) in HPV infected epithelial basal cells deregulates cell division [4]. Overexpression of these viral genes causes the deregulation of cell proliferation, metabolism, apoptosis, differentiation and genomic instability, all of which may lead to consecutive stages of cervical intra-epithelial neoplasia (CIN1, 2 and 3) or squamous intra-epithelial lesions (low-grade SIL and high-grade SIL). Approximately 85% of all sexually active persons will be exposed to HPV, and the majority of all HR-HPV infections are virion producing (which is limited in time) suggesting that in addition to the viral genotype, several cofactors are closely associated with the persistence of the viral oncoproteins and the transformation of the cervical mucosa to malignant tissue [5], [6].

There are several reasons why the development of a diagnostic assay, based on cervicovaginal fluid (CVF), for early detection of cervical cancer would be beneficial. At first, current screening assays are not optimal. Because of the high prevalence of cervical cancer, females between the ages of 25 and 65 are frequently screened using Pap(anicolaou) smears, a test which is based on detection of morphological changes of cervical mucosal cells. Unfortunately, the low sensitivity of this test can result in cervical cancer diagnosed at a late stage [7]. Data on randomized trials show that HPV DNA screening is more sensitive than cytology, but specificity is lower, which inevitably will result in an overtreatment because about 80% of the HPV positive patients spontaneously clear the virus [3]. Additionally, colposcopic examination is labor-intensive, difficult to automate and vulnerable to inter- and intra-observer variation [8]. The introduction of a new biomarker that helps improving sensitivity and specificity of current screening programs is therefore more than welcome. The second reason lies in the applicability of CVF for diagnosis. Because the fluid can easily be collected by the individual with the aid of special devices [9], a fast and simple test on the basis of a dipstick assay, performed by the woman herself, could be developed. Such a self test could be introduced e.g. in resource-limited populations where unequal burden of cervical cancer often occurs. Indeed, it is estimated that only 5% of women in low-resource countries are screened appropriately for cervical cancer [10]. Lack of healthcare infrastructure and financial cost are the main reasons why cytology-based programs are not implemented. Since alternative methods are currently investigated in these regions, use of a CVF-based self-diagnosis test could be considered. In addition, self tests could also be used in follow-up studies of vaccination programs since the effect of vaccination in sexually active women is uncertain [11]. Moreover, current vaccines only address neoplasia-inducing genotypes HPV16 and −18 suggesting that under ideal circumstances, only a maximum of 70% of all cervical cancers can be prevented [7], [12]. Thus at least for the coming decades, careful evaluation of vaccination programs is highly recommended and a simple self-diagnosis test could help a lot in meeting this question.

In the past, several proteomics studies were performed to identify biomarkers for the (early) detection of cervical neoplasia. In different studies, precancerous and healthy cervical tissue was compared and Lee *et al.* studied the *in vitro* contribution of oncoprotein E7 to the development of cervical neoplasia using a HPV-infected cell line [13]–[20]. Several markers that were linked to dysregulation of the normal cell cycle, such as p16, Ki67 and cyclin E were characterized. These findings resulted in improved understanding of HPV-induced carcinogenesis but so far they all showed low sensitivity and/or specificity and therefore could not be used in the clinic.

For biomarker discovery and validation, body fluids are better than tissue samples because the following advantages are inherent to most body fluids: (1) easy accessibility; (2) avoidance of sampling risks; and (3) multiple sampling potential [21]. Today, plasma is the most analyzed biological fluid in the search for biomarkers and it has already been used in cervical cancer research [22], [23]. However, plasma comes into contact with nearly all organs of the body, making plasma biomarkers less specific because it is difficult to determine from which organ they originate [21].

CVF can be obtained by cervicovaginal lavages, vaginal swabs, tampons or with a device for self-sampling [9], [24] and has the following benefits over plasma: (1) it possesses a lower fluid volume/organ-ratio compared to plasma, resulting in higher sensitivity; and (2) it comes into contact with fewer organ systems, which most likely increases the specificity of the markers [21], [25]. In this study, we focus on the identification of candidate biomarkers for cervical neoplasia by comparing the protein composition of individual CVF samples from healthy and precancerous (LSIL and HSIL) women. Our findings indicate that *alpha-actinin-4* (ACTN4) is a promising candidate biomarker for the precancerous state of cervical cancer and that this CVF protein is very well suited for the development of a self-diagnosis test.

## Materials and Methods

### Study design and sample collection

All patients agreed to participate by written consent and the study was approved by the ethical committee of the Antwerp university hospital (registration number: B30020108372). Study-specific patient identification codes were assigned and transmitted in such a manner that patient confidentiality was preserved.

All samples in this prospective, blinded, cohort study were taken by the same gynaecologist (WAAT). In case of abnormal Pap smear results (due to HPV infection or as a false positive outcome), both healthy and precancerous patients are routinely subjected to a colposcopic examination, a procedure that includes rinsing the vagina with 5% acetic acid. After colposcopy this washing fluid (containing the cervicovaginal fluid) is normally discarded but was collected for proteomic analysis. Additionally, cervical cytology samples were collected during this colposcopic examination to determine the cytology and HPV status as previously described by using type-specific PCRs [26]. All BD-SurePath liquid based cytology samples were sent to the pathology laboratory (RIATOL, Department of Molecular Diagnostics, Sonic Healthcare Benelux, Antwerp, Belgium) for processing. Based on the combination of colposcopic results, cytology outcome and HPV genotyping, healthy and precancerous women could be identified on a reliable manner. We selected samples originating from 6 healthy (normal colposcopy/cytology and HPV negative; group A) women and 6 precancerous (abnormal colposcopy/cytology and HR-HPV positive; group B) individuals (Table 1). All samples were derived from postmenopausal (> three years after the last menses) women from similar age (59+/−13 years). Patients who used medication were excluded from the study. Besides, patients who showed visible signs of gynaecological infections (e.g. gonorrhoea) or suffered a HIV-infection were excluded as well. Based on the cervicovaginal fluid samples, patients were screened for trichomonas vaginalis. During proteomic analyses, healthy and precancerous CVF samples were always analyzed in pairs to minimize technical variations of the LC-MS/MS platform.

**Table 1:** Patient information of samples used in the comprehensive proteomic experiments.

Sample	Age	Genotype(s)	Viral load	Colposcopy	Cytology	Unique Pept
A.1	57	*no HPV*		Normal	Normal	/
A.2	69	*no HPV*		Normal	Normal	/
A.3	52	*no HPV*		Normal	Normal	/
A.4	46	*no HPV*		Normal	Normal	/
A.5	66	*no HPV*		Normal	Normal	/
A.6	62	*no HPV*		Normal	Normal	/
B.1	61	*31/52*	335/2	LSIL	CIN1	4
B.2	53	*16*	417	HSIL	CIN3	2
B.3	47	*39*	2011	LSIL	CIN2	3
B.4	72	*18/56*	0.1322/1119	LSIL	CIN1	2
B.5	57	*31*	1148	HSIL	CIN3	12
B.6	60	*31/52*	258/25	LSIL	CIN2	6

Samples A1-6 are originating from healthy individuals (group A), while samples B1-6 originates from precancerous patients (group B). HPV viral load is expressed as number of copies per cell. Colposcopy: Normal  =  no aberrant lesions detected, LSIL  =  low-grade squamous intra-epithelial lesions, HSIL  =  high-grade squamous intra-epithelial lesions, ASCUS  =  atypical squamous cells of undetermined significance. Cytology is listed according to the Cervical Intraepithelial Neoplasia (CIN) nomenclature. The numbers of unique peptides of ACTN4 are listed in the last column.

### Proteomic analysis

After collection, the cervicovaginal lavages (25–40 ml) were immediately transported on ice to the laboratory and stored at −80°C. After centrifugation, the supernatant was concentrated by lyophilization to a final volume of approximately 200 µL. Protein samples (1 mg/sample) were separated and fractionated in the first dimension on a reverse phase (RP) protein C4 HPLC column. Enzymatic digestion with trypsin was performed and the resulting peptides of each fraction were separated in a second dimension on a RP-C18 micro-capillary HPLC system. Mass spectrometric analysis was performed using a MALDI-ToF/ToF instrument. Resulting MS/MS Spectra from each sample were screened against the human Swiss-Prot database (version: 57.1) using the MASCOT search engine. Analysis of the obtained datasets were performed as previously described [25]. More detailed information can be found in the Document S1 and Figure S1.

### Enzyme linked immunosorbent assay

To confirm the LC-MS/MS results, enzyme linked immune sorbent assays (ELISA) were performed for *alpha-actinin-4* (ACTN4) and *pyruvate kinase isozyme M1/M2* (PKM2), according to the manufacturer's instructions. A human ACTN4 ELISA kit (Cusabio Biotech Co. LTD.) and a human PKM2 ELISA kit (USCNK Life Science Inc.) were used to quantify ACTN4 and PKM2 in cervical vaginal washes from healthy (n = 16) and HPV-infected (n = 12) females (both pre- and post-menopausal). Additionally, several longitudinal samples (n = 29) were also tested for these two candidate biomarkers.

### Statistics

To determine whether the difference in protein abundances was significant, normalized spectra abundance factors (NSAF values) were subjected to statistical testing. Because the selected proteins were identified in several samples, and therefore also contained several NSAF-values, a non-parametric Mann-Whitney U-test was used. Moreover, a chi-square test was performed, whereby the frequency of all identified proteins was analyzed to determine whether certain proteins were more or exclusively detected more under certain conditions. Unpaired Student's T-tests were performed to analyze the ELISA results. All statistical tests were performed using the Statistical Package of Social Science (SPSS version 18).

## Results

### Differential study for the characterization of cervical cancer biomarkers

**Figure 1:**
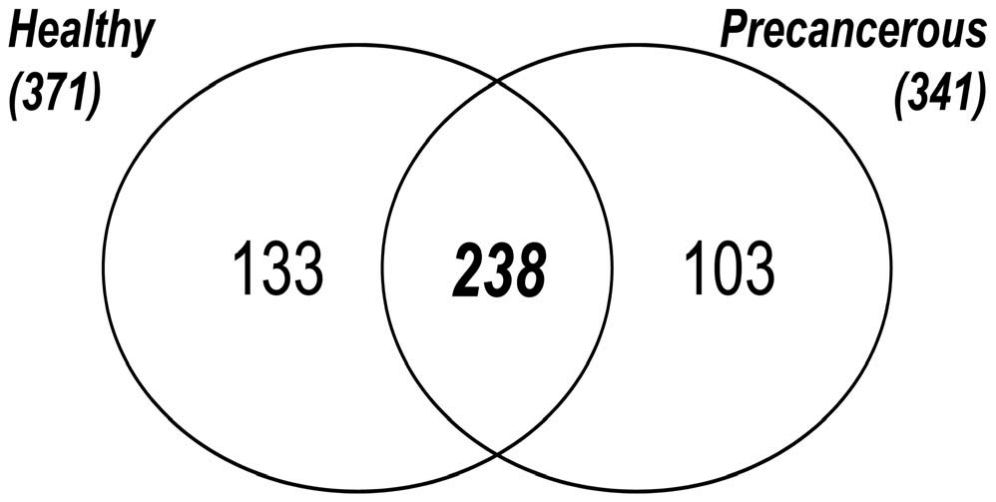
Venn diagram of the healthy and precancerous proteomes. For the healthy proteome 371 unique proteins were identified while 341 unique proteomes for the precancerous proteome. An overlapping set of 238 proteins were found in both proteomes.

To identify differentially abundant proteins, 12 samples were divided in two groups and were individually analyzed using an analytical proteomics platform (Table 1). Group A comprised of 6 samples from healthy individuals, whereas group B consisted of 6 samples from precancerous individuals. Proteomic analysis resulted in 846 and 825 identifications for group A and group B, respectively, representing 371 (group A) and 341 (group B) non-redundant proteins (Figure 1). The mean overlap between all samples from group A was 54% and from group B was 53% (Table S1).

A semi-quantitative analysis was also performed based on the label-free NSAF method (Table S2). For both groups, the NSAF values of the proteins present in at least 5 of the 6 samples were mutually compared. The variation in protein abundance between samples was expressed as the coefficient of variation (CV). A mean CV value of 60% (lowest: 19%; highest: 134%) was obtained for group A (healthy), while a mean CV of 52% (lowest: 14%; highest: 99%) was noted for group B.

### Identification of differentially abundant proteins

To identify candidate biomarkers that correlate with the precancerous state of cervical cancer, healthy and precancerous CVF proteomes were compared. A qualitative analysis was performed between the 371 identified proteins for the healthy group and the 341 identifications for the precancerous group, which resulted in 238 or 67% overlapping proteins. To identify proteins with a statistically significant difference of occurrence, all identifications were subjected to statistical testing using a Pearson chi-square test. Proteins with a p value ≤0.05 are listed (Table 2). From these calculations, ACTN4 was identified in all samples from precancerous patients but not in the samples from healthy patients. Moreover, ACTN4 was primarily identified based on several unique peptides with a mean MASCOT protein score of 162 (Table S3). The difference in the abundance of this protein was further verified by ELISA (see below).

**Table 2:** Proteins with a statistical significant difference in frequency between the healthy and precancerous group.

Acc.No	Protein	p-value	# Healthy	# Precanc
*O43707*	*Alpha-actinin-4*	*0,001*	*0*	*6*
P00558	Phosphoglycerate kinase 1	*0,014*	2	6
P02774	Vitamin D-binding protein precursor	*0,046*	6	3
P08603	Complement factor H	*0,046*	3	0
P29373	Cellular retinoic acid-binding protein 2	*0,046*	0	3
P29508	Squamous cell carcinoma antigen 1 (SCCA-1); Serpin B3	*0,046*	3	6
P43490	Nicotinamide phosphoribosyltransferase	*0,046*	0	3
P61158	Actin-related protein 3	*0,046*	0	3
P62258	14-3-3 protein epsilon	*0,014*	0	4
P62988	Ubiquitin	*0,014*	4	0
Q5VTE0	Putative elongation factor 1-alpha-like 3	*0,046*	3	6
Q9UIV8	Serpin B13	*0,046*	0	3

Acc No represents the accession number of the identified proteins. Statistical significance is listed in column named p-value. Columns “# Healthy” and “# Precanc” show the number of samples that contain the corresponding protein.

In addition, a semi-quantitative comparison was performed to determine whether certain proteins that were identified in both the healthy and the precancerous groups were differentially expressed. The NSAF-values of corresponding proteins were statistically tested using a Mann-Whitney U-test. Only proteins with a statistical significance of p≤0.05 are listed (Table 3). This calculation resulted in four proteins that were characterized by a significant difference in abundance between both conditions. Only one protein, *haptoglobin*, showed a downregulation in the precancerous condition, whereas three other proteins, *ATP synthase subunit beta*, *annexin A2* and PKM2, were upregulated. PKM2 and *annexin A2* showed the lowest p value (0.01 and 0.03, respectively) and were detected in 5 of 6 samples from the precancerous group. Because highly variable proteins are not suitable candidate biomarkers, the variation in the abundances of these proteins was calculated. With CV values of 40% and 29% for the healthy and precancerous group for PKM2, respectively, this protein is more suitable as a candidate biomarker compared to *annexin A2*, which had values of 77% and 48%, respectively.

**Table 3:** Proteins with a statistical significant difference in abundance between the healthy and precancerous group.

Acc.No	Protein	p-value	Mean Healthy	Mean Precanc.	Ratio
P00738	Haptoglobin	*0.03*	2,630 (3)	1,227 (5)	47%
P06576	ATP synthase subunit beta, mitochondrial	*0.05*	0,816 (3)	4,825 (3)	591%
P07355	Annexin A2	*0.03*	3,831 (6)	11,266 (5)	294%
*P14618*	*Pyruvate kinase isozymes M1/M2*	*0.01*	*0,452 (4)*	*1,401 (5)*	*310%*

Columns “Mean Healthy” and “Mean Precanc.” show the mean normalized spectral abundance factors (NSAF-value) while (x) indicates the number of samples that contain this ID. Ratio is expressed as precancerous/healthy. “Acc No” represents the accession number of the identified proteins and “p-value” shows the statistical significance.

### Verification of the candidate biomarkers

An ELISA was performed to verify the differential profiles of ACTN4 and PKM2. Healthy (n = 16) and HPV-infected samples (both HR-HPV oncogenic (n = 8) and LR-HPV non-oncogenic samples (n = 4)), which were not included in the previous experiment, were tested using commercially available kits (Table 4).

**Table 4:** Patient information of samples used for the validation of ACTN4 by ELISA.

Condition	Age	Genotype(s)	Viral load	Colposcopy	Cytology	Conc ACTN4
Healthy 1	54	*no HPV*		Normal	Normal	**85.6**
Healthy 2	53	*no HPV*		Normal	Normal	**1.1**
Healthy 3	41	*no HPV*		Normal	Normal	**12.2**
Healthy 4	21	*no HPV*		Normal	Normal	**3.7**
Healthy 5	50	*no HPV*		Normal	Normal	**14.4**
Healthy 6	52	*no HPV*		Normal	Normal	**1.0**
Healthy 7	59	*no HPV*		Normal	Normal	**6.2**
Healthy 8	34	*no HPV*		Normal	Normal	**7.2**
Healthy 9	36	*no HPV*		Normal	Normal	**−0.2**
Healthy 10	31	*no HPV*		Normal	Normal	**10.6**
Healthy 11	51	*no HPV*		Normal	Normal	**0.2**
Healthy 12	30	*no HPV*		Normal	Normal	**17.3**
Healthy 13	28	*no HPV*		Normal	Normal	**15.0**
Healthy 14	37	*no HPV*		Normal	Normal	**6.9**
Healthy 15	38	*no HPV*		Normal	Normal	**−13.0**
Healthy 16	62	*no HPV*		Normal	Normal	**16.3**
Low Risk 1	33	*6*	96249	ASCUS	Normal	**25.4**
Low Risk 2	37	*11*	51740	ASCUS	CIN1	**21.3**
Low Risk 3	42	*6*	1	Normal	Normal	**41.3**
Low Risk 4	49	*6*	0.05	Normal	Normal	**59.8**
High Risk 1	35	*16/39*	7729/1661	ASCUS	CIN3	**34.1**
High Risk 2	51	*52*	129	LSIL	CIN1	**227.8**
High Risk 3	20	*16/31/52/66*	0.02/11/12/31	LSIL	CIN1	**65.2**
High Risk 4	32	*16/31/39/52/66*	288/0.2/4/7416/179	LSIL	CIN1	**44.4**
High Risk 5	31	*16/58*	11571/253	HSIL	CIN2	**89.9**
High Risk 6	32	*16/53/58/59*	9126/79/2733/1510	LSIL	CIN1	**140.8**
High Risk 7	28	*35*	5159	LSIL	CIN2	**30.6**
High Risk 8	33	*31*	1696	HSIL	CIN1	**30.9**

HPV viral load was expressed as number of copies per cell. Colposcopy: Normal  =  no aberrant lesions detected, LSIL  =  low-grade squamous intra-epithelial lesions, HSIL  =  high-grade squamous intra-epithelial lesions, ASCUS  =  atypical squamous cells of undertimined significance. Cytology results were listed according to the Cervical Intraepithelial Neoplasia (CIN) nomenclature. The concentration of ACTN4 was described in pg/ml.

**Figure 2:**
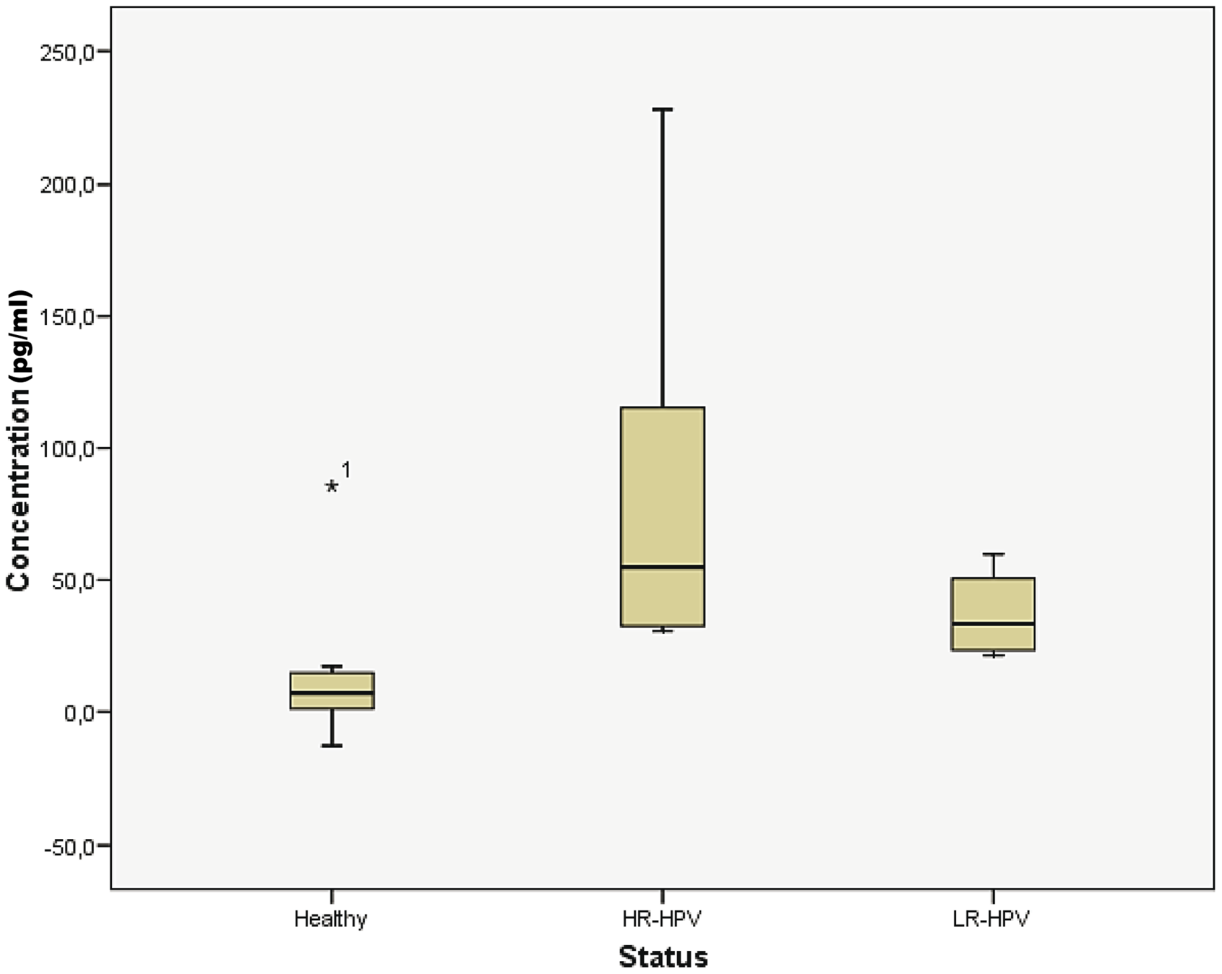
ACTN4 levels between different study groups. Concentration of ACTN4 for the healthy individuals (n = 16), the HR-HPV infected patients (n = 8) and the LR-HPV infected patients (n = 4). Statistical testing of ACNT-4 levels between healthy and HR-HPV infected patients resulted in a p-value of 0.023 and between healthy versus LR-HPV in a p-value of 0.052. When the HR- and LR-HPV infected patients were grouped as HPV infected patients, a p-value of 0.009 was found between non-infected (healthy patients) and infected groups.

Levels of ACTN4 were significantly higher in samples from HR-HPV infected individuals compared to samples from healthy, non-HR-HPV infected patients (p = 0.023), which confirmed our LC-MS/MS results. Remarkably, sample one, originating from a non-HR-HPV infected patient, appears to be an outlier because of the high ACTN4 concentration of 85.6 pg/mL (see discussion). Additionally, the LR-HPV infected patients had higher ACTN4 concentrations compared to the non-HPV-infected patients (p = 0.052), but somewhat lower values compared to the HR-HPV infected patients (p = 0.114). Comparison of the ACTN4 levels in HPV-infected (both HR- and LR-infections) versus non-infected samples (healthy patients) showed a significant difference, with a p value of 0.009 (Figure 2). The highest ACTN4 concentration for the healthy group (except for the outlier) was 17.3 pg/ml, whereas the lowest concentration for the HR-infected group was 30.6 pg/ml. The lowest concentration of ACTN4 in the LR-infected group was 21.3 pg/ml, which is higher than the highest value of the healthy group. Based on these observations, a threshold of 18 pg/ml can be set as the highest value of ACTN4 for the non-infected condition. No linear correlation was found between the viral load and the absolute concentration of ACTN4, which presumes that ACTN4 is not just a marker for HPV infection but rather for precancerous stage.

In addition, the most promising semi-quantitative candidate, PKM2, was verified with a commercial ELISA kit. For this analysis, the same 28 samples were used; however, the LC-MS/MS results were not confirmed (data not shown).

**Figure 3:**
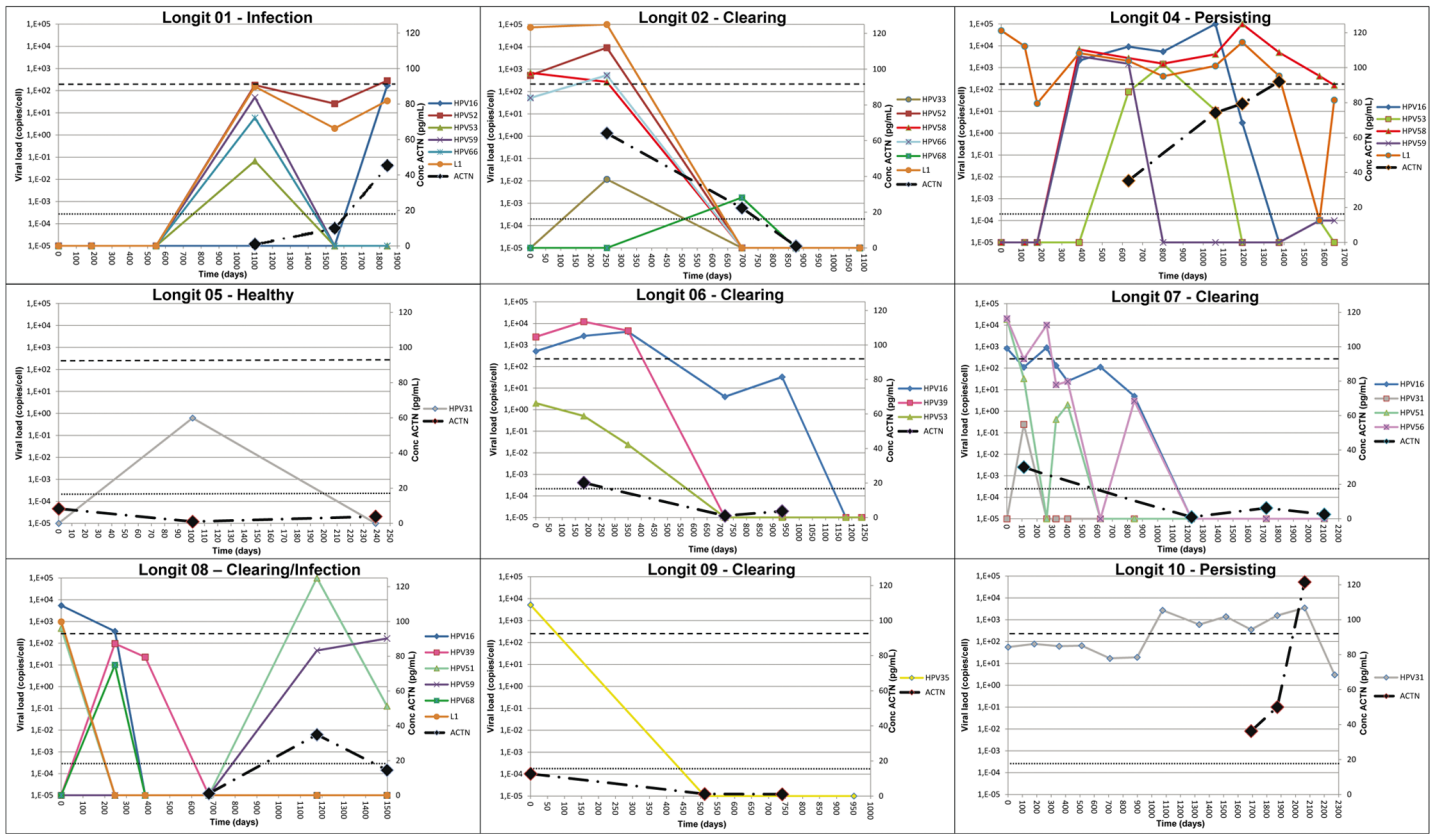
Evolution of ACTN4 levels and HPV infection on longitudinal samples. Comparison of ACTN4 levels with genotype and viral load in longitudinal samples from 9 different patients. Similar genotypes are illustrated in the same colours. At the left side the viral load (in number of copies/cell) is shown on a logarithmical scale while at the right side the concentration of ACTN4 is expressed in pg/ml. The y-axe indicates the time (expressed in days) while the patients were followed. A black dotted line illustrates the ACTN4 threshold of 17.3 pg/ml. ACTN4 levels were determined on the time points when a CVF sample was available.

We then analyzed the ACTN4 concentrations for an additional 29 longitudinal samples (i.e., samples from the same patient at several time points) from 9 patients. For each patient, a minimum of three longitudinal samples was evaluated (Figure 3). From these nine patients, five patients cleared the virus, two patients had persistent HPV infection, one patient acquired a new HPV infection and one patient was healthy. Although the numbers of patients per group are small, these samples can help us to confirm our findings. An ACTN4 ELISA was performed on several of the samples to identify correlations between the virus profiles and ACTN4 levels within one patient. However, in addition to the virus titer, we also expected the genotype and time of infection to influence the ACTN4 expression because different virus types have different carcinogenic capacities and cervical cancer usually develops after a persistent HR-HPV infection [2]. Recently, it was shown that the development of cervical precancer (cervical intraepithelial neoplasia of grade three; CIN3) is preceded by a steady increase in the viral load of a given HR-HPV type, whereas a rapid exponentially increasing load is generally cleared within 6 to 18 months and is usually associated with low-grade cytological abnormalities [27]. With these factors in consideration, the following two trends were observed from the experiments: (1) patients who had an early-stage infection (patient 1) or cleared the virus (patients 2, 6, 7 and 9) had increasing or descending ACTN4 levels, respectively, and patients with no or low infections (patient 5) or continuous infections (patients 4 and 10) had stable low or high ACTN4 levels, respectively; and (2) for a persistent infection (patients 1, 4 and 10), the ACTN4 accumulated.

## Discussion

### Characterizing the CVF proteome of healthy and precancerous patients

In this study the CVF proteome from 12 (6 healthy and 6 precancerous) individual samples was characterized to identify candidate protein biomarkers for the detection of the precancerous state of cervical cancer. The analysis of these 12 samples was performed at both the qualitative (present/absent) and semi-quantitative level. The number of protein identifications for each of the samples is very consistent with previously performed proteomic studies on CVF [28].

The determination of the amount of overlapping proteins between the samples within one group showed significant differences. Only 10% of all proteins were observed in each individual sample from both groups. This group consists primarily of proteins that are characteristic of the CVF proteome, based on their localization and biological function. We therefore call these proteins “characteristic proteins”. However, a significant number of identifications, such as intracellular proteins, are less characteristic of the CVF proteome. This finding can be explained by processes such as the disruption of the epithelial cell layer during sampling and the shedding of dead epithelial cells. We therefore group these identifications as “non-characteristic” proteins. Based on these findings, which are common to almost every comprehensive CVF proteomics study, one can make a distinction between a “core proteome” of frequently identified characteristic CVF proteins and a “variable proteome” that contains the non-characteristic CVF proteins [28].

Compared to the mean CV values of the common identifications between three technical replicates (25%) [25], the mean CV values of protein abundance in the healthy and the precancerous samples (60% and 52%, respectively) were significantly higher. This result was expected because, in contrast to the technical replicates, the samples were not identical. In all samples, proteins with high inter-individual differences were observed (up to 134%), whereas the measured mean CV value of the “characteristic proteins” was 38%. These results are similar to other proteomic studies of body fluids. For example, Yamakawa *et al.* [29] found a mean CV value of 63% for the seminal plasma proteome, and according to Anderson *et al.* [30], the mean CV value of a series of plasma proteins was approximately 45%.

### Identification of differentially abundant proteins

To examine differential abundant proteins, a label-free spectral counting method was performed. As this method allows only for a semi-quantitative analysis, outstanding effects were first investigated as these usually give the most reliable results. Therefore, we first pursued proteins for which their presence or absence in the precancerous group was characteristic for a given condition. Statistical testing (chi-square test) resulted in 12 proteins with a significant difference of incidence (p<0.05), of which 6 proteins were exclusively identified in precancerous samples (Table 2). The most significant protein was ACTN4 (Acc No: O43707), which was identified in all precancerous samples but none of the healthy samples (p = 0.001). Notably, SCCA-1, often used as plasma biomarker for cervical cancer [31], was also identified, albeit with a higher p value (p = 0.046).

ACTN4 is a critical component of the cytoskeleton and is involved in the formation of cell-cell adhesions [32]. It has been shown that the inhibition of certain membrane bound adhesion molecules caused by a malignant transformation results in a reduction of cell-cell adhesion strength and upregulation of ACTN4 [33]. Based on a knockdown study of ACTN4 using breast cancer cells, Khurana *et al.* demonstrated that ACTN4 was involved in the control mechanism of cell growth [34]. Moreover, the cytoplasmic accumulation of ACTN4 was associated with several malignancies and was linked with a bad prognosis [35], and an accumulative genomic gain of ACTN4 was associated with the formation of ovarian adenocarcinomas [36]. Also, RNAi-mediated downregulation of ACTN4 showed an association between the over expression of ACTN4 and an aggressive phenotype of oral squamous cell carcinoma [37]. Recently, the protein was found to be associated with several factors with varying functions, suggesting other roles for this protein, apart from actin regulation [38]. Indeed, Khurana *et al.* identified a LXXLL motif that was responsible for interaction of ACTN4 with the estrogen receptor and co-activators [39]. Recent studies in HPV transgenic mouse models provide evidence that estrogen and its nuclear receptor promote cervical cancer in combination with HPV oncogenes [40]. It is therefore tempting to speculate that ACTN4 plays a role in this important effect.

Based on the semi-quantitative NSAF-method, proteins were selected for their differential expression between the two conditions. Statistical testing (Mann-Whitney U) resulted in four proteins with a significant (p<0.05) difference in abundance (Table 3). Although these four proteins show a statistical significant difference during this discovery phase, a verification step is needed to confirm these results. When both the p-value and the frequency of presence were taken into account, *annexin A2* and PKM2 were most promising (p<0.03) because both were identified in at least 9 of 12 individual samples, with a three-fold upregulation for the precancerous condition (Table 3). Despite the identification of a*nnexin A2* in nearly every sample (11/12 samples) with an acceptable difference in abundance (p = 0.03), the inter-individual variations (CV 77%) for the healthy group were much higher compared to PKM2 (35%). Therefore, PKM2 (Acc No: P14618) was selected for verification by ELISA.

PKM2 is a glycolytic enzyme that contributes to the energy supply of the cell. This protein has two isoforms, an embryological isoform (M2) with elevated enzymatic activity and an adult isoform (M1). Recently, it was demonstrated that malignant transformations of cells by HR-HPV oncogenes E6 and E7 induced a PKM switch from M1 to M2 [41]. This switch causes a shift from normal cellular metabolism to elevated glycolysis, which is beneficial for the growth and neoplasm of tumor cells [42]. Unfortunately, we were not able to confirm the difference in PKM2 concentrations between CVFs from healthy and precancerous individuals, although two ELISA kits from different suppliers were used. It is possible that the semi-quantitative spectral counting method may be prone to too much variation. Alternatively, we have previously observed that the CVF matrix is not optimal for some ELISA configurations and this may be the case for the two PKM2 ELISA assays.

### Verification of alpha-actinin-4 levels

Alpha-actinin-4, the most promising candidate biomarker, was verified on 57 additional CVF samples (28 individual samples and 29 longitudinal samples) by ELISA, a number that certainly fulfills the requirements for the discovery/verification phase of protein biomarkers [43]. The ELISA results showed that the concentration of ACTN4 is elevated in the HPV infected patients compared to the non-infected patients, thereby confirming the LC-MS/MS results. However, infection with LR-HPV (HPV-6 and HPV-11) resulted in higher ACTN4 concentrations in the CVF. Although the concentrations of ACTN4 measured in the CVFs from LR-HPV-infected individuals are higher than those from non-infected individuals, no significant difference in the abundance level was observed (p = 0.052). Because these findings are based on the analysis of samples from only four single LR-HPV-infected patients, more of this type of sample must be evaluated before conclusions can be drawn. One non-infected patient showed an outlier value of 85.6 pg/ml of ACTN4. This patient did not have a known history of HR-HPV infection, but the clinical file showed ischemic heart disease and a prevalence of breast cancer. It is therefore possible that this elevated concentration of ACTN4 is caused by the medical treatment or an LR-HPV infection other than HPV-6 or HPV-11. Therefore, extensive validation of this marker on a larger number of samples, including those from patients suffering from other diseases and infections (HIV, herpes, bacterial vaginosis, *Chlamydia* etc.), is the next step to determine whether ACTN4 is suitable for diagnosis purposes. In these studies, one could set a threshold at 18 pg/ml to discriminate between samples from healthy versus HPV-infected individuals.

Although ACTN4 levels did not numerically correlate with the viral loads, an ascending and descending trend could clearly be observed in patients who either started an infection or cleared the virus. We also observed that infections over longer periods caused accumulation of the marker. A high level of ACTN4 may therefore be an indication of a persistent oncogenic HPV infection and an increased chance of developing cervical cancer [6], [44]. It was generally observed in longitudinal samples that total viral loads of approximately 100 particles/cell caused ACTN4 levels above the threshold value of 18 pg/ml. An exception on this rule was observed in patient 9, in which a viral load of 5,159 HPV35 particles/cell resulted in only 12.7 pg/ml ACTN4 (Figure 3). Whether this false negative result can be explained by the very low oncogenic potential of HPV35 must be investigated [2]. Conversely, two patients infected with very low doses of HPV6 had exceptionally high levels of ACTN4 (Table 4). These false positives could be the result of an exceptionally high ACTN4-inducing capacity by HPV6 or of an additional viral, fungal, or bacterial infection that also caused an increase of ACTN4 levels in the CVF. One must be aware however, that release of ACTN4 is possibly a consequence of an immune-induced lysis of a precancerous lesions, since HPV virions are shed into the environment in the absence of lysis or necrosis [45]. This suggests that HPV virus production and ACTN4 release do not necessarily occur simultaneously, although they usually do. In the two above mentioned patients however, virus production may have stopped while precancerous lesions or benign tumors continue to be target for the crippled immune system, resulting in a concomitant release of ACTN4. Further validation steps, on a larger amount of samples, are required to determine the specificity and sensitivity of ACTN4 for the presence of HPV and/or precancerous lesions. Nevertheless, these results suggest that the HPV genotype, duration of infection and immune reaction may determine the abundance of ACTN4 in the CVF.

### Application and future perspectives

Although earlier screening programs have undoubtedly reduced mortality and morbidity caused by cervical cancer, the characterization of one or more proteins that correlate with HPV infection and/or the pre-malignant state will further improve the efficiency of the current screening methods for cervical cancer [8]. As such, false positive and false negative diagnoses will be decreased such that gynaecologists can focus at risk patients. But in addition to diagnostic improvement, CVF protein biomarkers such as ACTN4, allow for the development of relatively simple antibody-based tests (‘dipstick’). A significant portion of the female population does not participate in any screening, especially those who live in low resource countries, and many of these women would be willing to use a self-sampling test or use self-sampling devices for CVF collection [9],[46],[47].

## Supporting Information

Figure S1 **Calibration curve for RP-C4 protein quantification.**
(JPG)

Table S1 **Overlap of identified proteins.**
(DOC)

Table S2 **List of identified proteins and NSAF values.**
(XLS)

Table S3 **List of peptides involved in alpha-actinin-4 identification and quantification.**
(XLS)

Document S1 **Additional Information on Material and Methods.**
(DOC)

## References

[1] Snijders PJ, Steenbergen RD, Heideman DA, Meijer CJ (2006) HPV-mediated cervical carcinogenesis: concepts and clinical implications. J Pathol 208: 152–164 10.1002/path.1866 [doi]. 16362994 10.1002/path.1866

[2] Sjoeborg KD, Trope A, Lie AK, Jonassen CM, Steinbakk M, et al (2010) HPV genotype distribution according to severity of cervical neoplasia. Gynecol Oncol 118: 29–34 S0090-8258(10)00257-X [pii];10.1016/j.ygyno.2010.03.007 [doi]. 20406711 10.1016/j.ygyno.2010.03.007

[3] Paavonen J (2007) Human papillomavirus infection and the development of cervical cancer and related genital neoplasias. Int J Infect Dis 11 Suppl 2S3–S9 S1201-9712(07)60015-0 [pii];10.1016/S1201-9712(07)60015-0 [doi]. 18162244 10.1016/S1201-9712(07)60015-0

[4] Maglennon GA, McIntosh P, Doorbar J (2011) Persistence of viral DNA in the epithelial basal layer suggests a model for papillomavirus latency following immune regression. Virology 414: 153–163 S0042-6822(11)00144-9 [pii];10.1016/j.virol.2011.03.019 [doi]. 21492895 10.1016/j.virol.2011.03.019 PMC3101335

[5] Woodman CB, Collins SI, Young LS (2007) The natural history of cervical HPV infection: unresolved issues. Nat Rev Cancer 7: 11–22 nrc2050 [pii];10.1038/nrc2050 [doi]. 17186016 10.1038/nrc2050

[6] Kjaer SK, van den Brule AJ, Paull G, Svare EI, Sherman ME, et al (2002) Type specific persistence of high risk human papillomavirus (HPV) as indicator of high grade cervical squamous intraepithelial lesions in young women: population based prospective follow up study. BMJ 325: 572. 12228133 10.1136/bmj.325.7364.572 PMC124551

[7] Einstein MH, Schiller JT, Viscidi RP, Strickler HD, Coursaget P, et al (2009) Clinician's guide to human papillomavirus immunology: knowns and unknowns. Lancet Infect Dis 9: 347–356 S1473-3099(09)70108-2 [pii];10.1016/S1473-3099(09)70108-2 [doi]. 19467474 10.1016/S1473-3099(09)70108-2

[8] Schiffman M, Wentzensen N, Wacholder S, Kinney W, Gage JC, et al (2011) Human papillomavirus testing in the prevention of cervical cancer. J Natl Cancer Inst 103: 368–383 djq562 [pii];10.1093/jnci/djq562 [doi]. 21282563 10.1093/jnci/djq562 PMC3046952

[9] Gok M, Heideman DA, van Kemenade FJ, Berkhof J, Rozendaal L, et al (2010) CJ (2010) HPV testing on self collected cervicovaginal lavage specimens as screening method for women who do not attend cervical screening: cohort study. BMJ 340: c1040. 20223872 10.1136/bmj.c1040 PMC2837143

[10] Bradford L, Goodman A (2013) Cervical cancer screening and prevention in low-resource settings. Clin Obstet Gynecol 56: 76–87 10.1097/GRF.0b013e31828237ac [doi]. 23337844 10.1097/GRF.0b013e31828237ac

[11] Trottier H, Ferreira S, Thomann P, Costa MC, Sobrinho JS, et al (2010) Human papillomavirus infection and reinfection in adult women: the role of sexual activity and natural immunity. Cancer Res 70: 8569–8577 0008-5472.CAN-10-0621 [pii];10.1158/0008-5472.CAN-10-0621 [doi]. 20978200 10.1158/0008-5472.CAN-10-0621 PMC4068337

[12] Crosignani P, De SA, Fara GM, Isidori AM, Lenzi A, et al (2013) Towards the eradication of HPV infection through universal specific vaccination. BMC Public Health 13: 642 1471-2458-13-642 [pii];10.1186/1471-2458-13-642 [doi]. 23845195 10.1186/1471-2458-13-642 PMC3751659

[13] Zhu X, Lv J, Yu L, Zhu X, Wu J, et al (2009) Proteomic identification of differentially-expressed proteins in squamous cervical cancer. Gynecol Oncol 112: 248–256 S0090-8258(08)00809-3 [pii];10.1016/j.ygyno.2008.09.045 [doi]. 19007971 10.1016/j.ygyno.2008.09.045

[14] Bae SM, Lee CH, Cho YL, Nam KH, Kim YW, et al (2005) Two-dimensional gel analysis of protein expression profile in squamous cervical cancer patients. Gynecol Oncol 99: 26–35 S0090-8258(05)00390-2 [pii];10.1016/j.ygyno.2005.05.041 [doi]. 16051329 10.1016/j.ygyno.2005.05.041

[15] Wong YF, Cheung TH, Lo KW, Wang VW, Chan CS, et al (2004) Protein profiling of cervical cancer by protein-biochips: proteomic scoring to discriminate cervical cancer from normal cervix. Cancer Lett 211: 227–234 10.1016/j.canlet.2004.02.014 [doi];S0304383504001673 [pii]. 15219946 10.1016/j.canlet.2004.02.014

[16] Choi YP, Kang S, Hong S, Xie X, Cho NH (2005) Proteomic analysis of progressive factors in uterine cervical cancer. Proteomics 5: 1481–1493 10.1002/pmic.200401021 [doi]. 15838902 10.1002/pmic.200401021

[17] Uleberg KE, Munk AC, Skaland I, Furlan C, Van Diermen B, et al.. (2011) A protein profile study to discriminate CIN lesions from normal cervical epithelium. Cell Oncol (Dordr). 10.1007/s13402-011-0047-3 [doi]. 10.1007/s13402-011-0047-3 PMC3219864 21573931

[18] Arnouk H, Merkley MA, Podolsky RH, Stoppler H, Santos C, et al (2009) Characterization of Molecular Markers Indicative of Cervical Cancer Progression. Proteomics Clin Appl 3: 516–527 10.1002/prca.200800068 [doi]. 19834583 10.1002/prca.200800068 PMC2761690

[19] Lomnytska MI, Becker S, Hellman K, Hellstrom AC, Souchelnytskyi S, et al (2010) Diagnostic protein marker patterns in squamous cervical cancer. Proteomics Clin Appl 4: 17–31 10.1002/prca.200900086 [doi]. 21137014 10.1002/prca.200900086

[20] Lee KA, Kang JW, Shim JH, Kho CW, Park SG, et al (2005) Protein profiling and identification of modulators regulated by human papillomavirus 16 E7 oncogene in HaCaT keratinocytes by proteomics. Gynecol Oncol 99: 142–152 S0090-8258(05)00411-7 [pii];10.1016/j.ygyno.2005.05.039 [doi]. 16038965 10.1016/j.ygyno.2005.05.039

[21] Good DM, Thongboonkerd V, Novak J, Bascands JL, Schanstra JP, et al (2007) Body fluid proteomics for biomarker discovery: lessons from the past hold the key to success in the future. J Proteome Res 6: 4549–4555 10.1021/pr070529w [doi]. 17970587 10.1021/pr070529w

[22] Lin YW, Lai HC, Lin CY, Chiou JY, Shui HA, et al (2006) Plasma proteomic profiling for detecting and differentiating in situ and invasive carcinomas of the uterine cervix. Int J Gynecol Cancer 16: 1216–1224 IJG583 [pii];10.1111/j.1525-1438.2006.00583.x [doi]. 16803509 10.1111/j.1525-1438.2006.00583.x

[23] Guo X, Abliz G, Reyimu H, Zhao F, Kadeer N, et al (2012) The association of a distinct plasma proteomic profile with the cervical high-grade squamous intraepithelial lesion of Uyghur women: a 2D liquid-phase chromatography/mass spectrometry study. Biomarkers 17: 352–361 10.3109/1354750X.2012.673133 [doi]. 22458349 10.3109/1354750X.2012.673133

[24] Brink AA, Meijer CJ, Wiegerinck MA, Nieboer TE, Kruitwagen RF, et al (2006) High concordance of results of testing for human papillomavirus in cervicovaginal samples collected by two methods, with comparison of a novel self-sampling device to a conventional endocervical brush. J Clin Microbiol 44: 2518–2523 44/7/2518 [pii];10.1128/JCM.02440-05 [doi]. 16825374 10.1128/JCM.02440-05 PMC1489519

[25] Zegels G, Van Raemdonck GA, Coen EP, Tjalma WA, Van Ostade XW (2009) Comprehensive proteomic analysis of human cervical-vaginal fluid using colposcopy samples. Proteome Sci 7: 17 1477-5956-7-17 [pii];10.1186/1477-5956-7-17 [doi]. 19374746 10.1186/1477-5956-7-17 PMC2678104

[26] Depuydt CE, Boulet GA, Horvath CA, Benoy IH, Vereecken AJ, et al (2007) Comparison of MY09/11 consensus PCR and type-specific PCRs in the detection of oncogenic HPV types. J Cell Mol Med 11: 881–891 JCMM073 [pii];10.1111/j.1582-4934.2007.00073.x [doi]. 17760847 10.1111/j.1582-4934.2007.00073.x PMC3823264

[27] Depuydt CE, Criel AM, Benoy IH, Arbyn M, Vereecken AJ, et al (2012) Changes in type-specific human papillomavirus load predict progression to cervical cancer. J Cell Mol Med 16: 3096–3104 10.1111/j.1582-4934.2012.01631.x [doi]. 22978795 10.1111/j.1582-4934.2012.01631.x PMC4393737

[28] Zegels G, Van Raemdonck GA, Tjalma WA, Van Ostade XW (2010) Use of cervicovaginal fluid for the identification of biomarkers for pathologies of the female genital tract. Proteome Sci 8: 63 1477-5956-8-63 [pii];10.1186/1477-5956-8-63 [doi]. 21143851 10.1186/1477-5956-8-63 PMC3016264

[29] Yamakawa K, Yoshida K, Nishikawa H, Kato T, Iwamoto T (2007) Comparative analysis of interindividual variations in the seminal plasma proteome of fertile men with identification of potential markers for azoospermia in infertile patients. J Androl 28: 858–865 jandrol.107.002824 [pii];10.2164/jandrol.107.002824 [doi]. 17554110 10.2164/jandrol.107.002824

[30] Anderson NL, Anderson NG (2002) The human plasma proteome: history, character, and diagnostic prospects. Mol Cell Proteomics 1: 845–867. 12488461 10.1074/mcp.r200007-mcp200

[31] Gadducci A, Tana R, Cosio S, Genazzani AR (2008) The serum assay of tumour markers in the prognostic evaluation, treatment monitoring and follow-up of patients with cervical cancer: a review of the literature. Crit Rev Oncol Hematol 66: 10–20 S1040-8428(07)00183-7 [pii];10.1016/j.critrevonc.2007.09.002 [doi]. 17964182 10.1016/j.critrevonc.2007.09.002

[32] Nakatsuji H, Nishimura N, Yamamura R, Kanayama HO, Sasaki T (2008) Involvement of actinin-4 in the recruitment of JRAB/MICAL-L2 to cell-cell junctions and the formation of functional tight junctions. Mol Cell Biol 28: 3324–3335 MCB.00144-08 [pii];10.1128/MCB.00144-08 [doi]. 18332111 10.1128/MCB.00144-08 PMC2423145

[33] Barbolina MV, Adley BP, Kelly DL, Fought AJ, Scholtens DM, et al (2008) Motility-related actinin alpha-4 is associated with advanced and metastatic ovarian carcinoma. Lab Invest 88: 602–614 labinvest200825 [pii];10.1038/labinvest.2008.25 [doi]. 18362906 10.1038/labinvest.2008.25 PMC2849305

[34] Khurana S, Chakraborty S, Cheng X, Su YT, Kao HY (2011) The actin-binding protein, actinin alpha 4 (ACTN4), is a nuclear receptor coactivator that promotes proliferation of MCF-7 breast cancer cells. J Biol Chem 286: 1850–1859 M110.162107 [pii];10.1074/jbc.M110.162107 [doi]. 21078666 10.1074/jbc.M110.162107 PMC3023480

[35] Yamamoto S, Tsuda H, Honda K, Onozato K, Takano M, et al (2009) Actinin-4 gene amplification in ovarian cancer: a candidate oncogene associated with poor patient prognosis and tumor chemoresistance. Mod Pathol 22: 499–507 modpathol2008234 [pii];10.1038/modpathol.2008.234 [doi]. 19151661 10.1038/modpathol.2008.234

[36] Yamamoto S, Tsuda H, Honda K, Takano M, Tamai S, et al (2012) ACTN4 gene amplification and actinin-4 protein overexpression drive tumour development and histological progression in a high-grade subset of ovarian clear-cell adenocarcinomas. Histopathology 60: 1073–1083 10.1111/j.1365-2559.2011.04163.x [doi]. 22348389 10.1111/j.1365-2559.2011.04163.x

[37] Yamada S, Yanamoto S, Yoshida H, Yoshitomi I, Kawasaki G, et al (2010) RNAi-mediated down-regulation of alpha-actinin-4 decreases invasion potential in oral squamous cell carcinoma. Int J Oral Maxillofac Surg 39: 61–67 S0901-5027(09)01125-4 [pii];10.1016/j.ijom.2009.10.003 [doi]. 19913389 10.1016/j.ijom.2009.10.003

[38] Foley KS, Young PW (2013) An analysis of splicing, actin-binding properties, heterodimerization and molecular interactions of the non-muscle alpha-actinins. Biochem J 452: 477–488 BJ20121824 [pii];10.1042/BJ20121824 [doi]. 23557398 10.1042/BJ20121824

[39] Khurana S, Chakraborty S, Zhao X, Liu Y, Guan D, et al (2012) Identification of a novel LXXLL motif in alpha-actinin 4-spliced isoform that is critical for its interaction with estrogen receptor alpha and co-activators. J Biol Chem 287: 35418–35429 M112.401364 [pii];10.1074/jbc.M112.401364 [doi]. 22908231 10.1074/jbc.M112.401364 PMC3471738

[40] Chung SH, Franceschi S, Lambert PF (2010) Estrogen and ERalpha: culprits in cervical cancer? Trends Endocrinol Metab 21: 504–511 S1043-2760(10)00062-7 [pii];10.1016/j.tem.2010.03.005 [doi]. 20456973 10.1016/j.tem.2010.03.005 PMC2914219

[41] Christofk HR, Vander Heiden MG, Harris MH, Ramanathan A, Gerszten RE, et al (2008) The M2 splice isoform of pyruvate kinase is important for cancer metabolism and tumour growth. Nature 452: 230–233 nature06734 [pii];10.1038/nature06734 [doi]. 18337823 10.1038/nature06734

[42] Mazurek S (2007) Pyruvate kinase type M2: a key regulator within the tumour metabolome and a tool for metabolic profiling of tumours. Ernst Schering Found Symp Proc 99–124. 10.1007/2789_2008_091 18811055

[43] Parker CE, Borchers CH (2014) Mass spectrometry based biomarker discovery, verification, and validation - Quality assurance and control of protein biomarker assays. Mol Oncol 8: 840–858 S1574-7891(14)00054-4 [pii];10.1016/j.molonc.2014.03.006 [doi]. 24713096 10.1016/j.molonc.2014.03.006 PMC5528535

[44] Bodily J, Laimins LA (2011) Persistence of human papillomavirus infection: keys to malignant progression. Trends Microbiol 19: 33–39 S0966-842X(10)00177-0 [pii];10.1016/j.tim.2010.10.002 [doi]. 21050765 10.1016/j.tim.2010.10.002 PMC3059725

[45] Stanley M (2008) Immunobiology of HPV and HPV vaccines. Gynecol Oncol 109: S15–S21 S0090-8258(08)00105-4 [pii];10.1016/j.ygyno.2008.02.003 [doi]. 18474288 10.1016/j.ygyno.2008.02.003

[46] Cerigo H, Coutlee F, Franco EL, Brassard P (2012) Dry self-sampling versus provider-sampling of cervicovaginal specimens for human papillomavirus detection in the Inuit population of Nunavik, Quebec. J Med Screen 19: 42–48 19/1/42 [pii];10.1258/jms.2012.012011 [doi]. 10.1258/jms.2012.012011 22438506

[47] Dzuba IG, Diaz EY, Allen B, Leonard YF, Lazcano Ponce EC, et al (2002) The acceptability of self-collected samples for HPV testing vs. the pap test as alternatives in cervical cancer screening. J Womens Health Gend Based Med 11: 265–275 10.1089/152460902753668466 [doi]. 11988136 10.1089/152460902753668466

