# Supplementary figures and images for: Identification of Protein Biomarkers for Cervical Cancer Using Human Cervicovaginal Fluid
Source: PLoS One. 2014 Sep 12;9(9):e106488. doi: 10.1371/journal.pone.0106488 (PMC4162552; doi:10.1371/journal.pone.0106488)

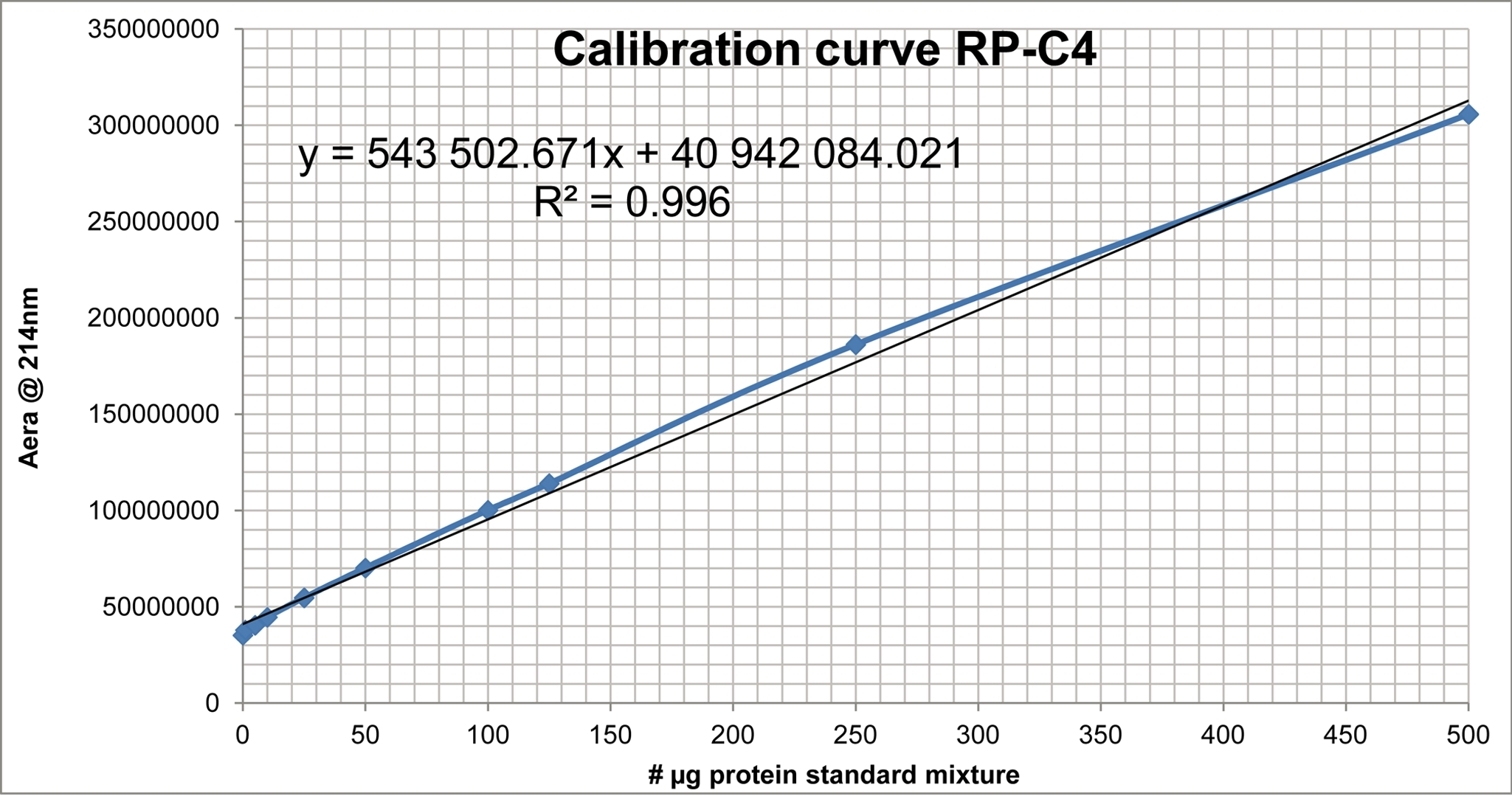

Supplement: Figure S1 — Calibration curve for RP-C4 protein quantification. (JPG) [file pone.0106488.s001.jpg]
