# Supplementary material for: Identification of Protein Biomarkers for Cervical Cancer Using Human Cervicovaginal Fluid
Source: PLoS One. 2014 Sep 12;9(9):e106488. doi: 10.1371/journal.pone.0106488 (PMC4162552; doi:10.1371/journal.pone.0106488)
Supplement: Table S1 — Overlap of identified proteins. (DOC) [file pone.0106488.s002.doc]

**Table S1. Percentage overlap of the identified proteins for each analyzed sample.**

|  | H.1 | H.2 | H.3 | H.4 | H.5 | H.6 |  | P.1 | P.2 | P.3 | P.4 | P.5 | P.6 |
| --- | --- | --- | --- | --- | --- | --- | --- | --- | --- | --- | --- | --- | --- |
| H.1 | **100%** |  |  |  |  |  | P.1 | **100%** |  |  |  |  |  |
| H.2 | 67% | **100%** |  |  |  |  | P.2 | 59% | **100%** |  |  |  |  |
| H.3 | 65% | 63% | **100%** |  |  |  | P.3 | 56% | 47% | **100%** |  |  |  |
| H.4 | 63% | 69% | 61% | **100%** |  |  | P.4 | 62% | 54% | 56% | **100%** |  |  |
| H.5 | 60% | 58% | 52% | 55% | **100%** |  | P.5 | 52% | 48% | 50% | 57% | **100%** |  |
| H.6 | 40% | 44% | 38% | 41% | 36% | **100%** | P.6 | 57% | 55% | 36% | 48% | 55% | **100%** |
