# Supplementary material for: Identification of Protein Biomarkers for Cervical Cancer Using Human Cervicovaginal Fluid
Source: PLoS One. 2014 Sep 12;9(9):e106488. doi: 10.1371/journal.pone.0106488 (PMC4162552; doi:10.1371/journal.pone.0106488)
Supplement: Document S1 — Additional Information on Material and Methods. (DOC) [file pone.0106488.s005.doc]

**Document S1. Detailed information about the experimental setup and used materials and methods.**

*Sample preparation for proteomic analysis*

After collection, the cervical-vaginal lavages (25-40ml) were immediately transported on ice to the laboratory and stored at -80°C. All samples were centrifuged at 4°C for 150 min at 10,000 g (Sorvall SC-500 plus, fixed-angle rotor) to remove cells and debris. The supernatant was concentrated by lyophilization using a Savant Speed-Vac SC-100 instrument equipped with an Edwards vacuum pump, until a final volume of approximately 200µL was obtained. To determine the protein concentration, a small fraction of the lyophilized sample (20 µL) was loaded onto a reverse phase C4 high performance liquid chromatographic (HPLC) system (see below). Based on the area under the curve (AUC) at 214nm, an estimation of the protein amount could be calculated by using the equation of a standard curve (Supporting information S5). The mean protein concentration of the CVF samples was 3513 ± 2703 µg/mL. No significant differences were found between the healthy and the precancerous samples.

*Chromatographic separation and protein digestion*

Concentrated protein samples (1 mg/sample) were separated and fractionated in the first dimension on a reverse phase (RP) protein C4 VYDAC HPLC column (214TP5415; 4.6 x 150 mm, particle size 5 µm; Alltech Associates Inc., Lokeren, Belgium) using a Waters 600-series HPLC system (Waters Corporation, Milford, Ma, USA). Proteins were separated on the RP-C4 column using the following gradient: 3 to 15% B over 5 min, from 15 to 60% B over 15 min, from 60 to 100% B over 3 min, 100% B for 2 min and back to 3% B over 3 min. Solvent A was composed of water with 0.1% TFA, while solvent B was composed of acetonitrile with 0.1% TFA. In all cases, 14 fractions were collected from 4 to 25 min (1.5 ml/fraction). Before digestion, all fractions were lyophilized to dryness. Fractions with lowest protein concentrations were pooled, after which 10 fractions remained. Finally, proteomics-grade modified trypsin (Roche, Mannheim, Germany) was added at a 30:1 protein-to-enzyme ratio. After incubation at 37°C for 18 h, the digest was stopped by freezing the fractions at -80°C. Peptide fractions (10µg per fraction) were separated in a second dimension on a micro-capillary RP-C18 column (Zorbax 300SB-C18; 0.3mm x 150mm; 3.5µm particle size; Agilent Technologies, Waldbronn, Germany) using an Agilent 1100 series micro-capillary HPLC system. Peptides were separated on the micro-capillary RP-C18 column using the following gradient: 5 to 55% B over 51.70 min, from 55 to 90% B over 8.30 min, 100% B for 5 min and back to 5% B over 5 min. Solvent A was composed of water with 0.1% FA, while solvent B was composed of 90% acetonitrile with 0.1% FA. Three hundred fifty spots (800 nl/spot) for each fraction were spotted on an Opti-TOF MALDI-target (28 columns x 25 rows; 8 sec interval; two runs per target) (Applied Biosystems Inc., Forester City, CA, USA). Afterwards, each spot was covered with matrix (2 mg/ml α-cyano-4-hydroxycinnamic acid in 70% ACN; internal calibrant: 63 pmol/ml human [Glu^1^]-fibrinopeptide B).

*Mass spectrometric analysis*

Spotted fractions were analyzed using a matrix assisted laser desorption/ionization (MALDI) AB4800 proteomics analyzer (Applied Biosystems). MALDI-ToF MS-analysis (reflectron mode; laser intensity: 3400; 25 x 20 laser shots per spot; mass-range 700-2500 Da) was performed first, after which precursors with a signal-to-noise (S/N) ratio above or equal to 100 were selected. MALDI-ToF/ToF MS/MS-analysis was performed on these selected precursors, and a maximum of 50 unique precursors per spot were selected for fragmentation, beginning with the precursors with the lowest S/N-ratio. These precursors were ionized (laser intensity: 4400; 25 x 20 laser shots per spot) and fragmented in a collision cell (1kV collision) with air.

*Data analysis*

Spectra from each sample were screened against the human Swiss-Prot database (version: 57.1) using the MASCOT search engine (Matrix Science; version 2.1.03) and GPS Explorer Software (Applied Biosystems). Carbamidomethylation of cysteines was listed as fixed modification, and oxidation of methionine was listed as a variable modification. A maximum of two missed cleavages of trypsin was tolerated. The mass tolerance was set to 200 ppm for the precursors and 0.2 Da for the fragment ions. The MudPIT scoring algorithm of MASCOT was used. All keratins were excluded from the protein lists.

To determine whether the applied identification methodology was sufficiently stringent, the false discovery rate (FDR) of the protein level was estimated using a concatenated database consisting of the target Swiss-Prot database and a shuffled Swiss-Prot database. Calculation of the FDR was performed as follows: 2x false positive identifications divided by false positive identifications plus true positive identifications [1]. The MS/MS spectra were screened at protein level against a concatenated database to determine the FDR, and all samples resulted in ratios equal to or less than 5%.

*Semi-quantitative analysis*

The abundance of each identified protein was estimated using a semi-quantitative spectral counting (SC) method. SC is based on the finding that the number of validated MS/MS spectra is a benchmark for the relative abundance of this protein [2-4]. The total count of MS/MS spectra was determined for each protein. To correct the obtained spectral counts for the differences in protein size, a normalization step was performed by dividing the number of counted spectra with the number of predicted observable peptides [3, 5]. The observable peptides were determined by an *in silico* tryptic digest of the corresponding protein using the freeware tool “Peptide Mass” (http://web.expasy.org/peptide_mass/). However, not all the *in silico* generated peptides are detectable because of technical limitations of the LC-MS platform. Therefore, all *in silico* tryptic peptides were filtered according to their mass (mass range MS: 700-2,500 Da) and retention time (min 5 – 51.7) using the “Sequence Specific Retention Calculator” version 3.0 [6], after which, only the observable peptides remained. For the semi-quantitative analysis of the identified proteins, the total spectral counts of the MS/MS spectra were calculated for each protein using the normalized spectral abundance factor (NSAF), according to the following equation [2]:

$$(NSAF)_{k}=\left(\frac{(SC/OP)_{k}}{\sum_{i=1}^{N} (SC/OP)_{i}}\right)\times1000$$

where SC is the number of spectral counts for protein k, OP is the number of observable peptides and N is the total number of identified proteins in one experiment. These values were multiplied by a factor of 1,000 for convenience. To compare protein abundances based on the NSAF values, Microsoft® Office Access 2007 was used to create cross-tables (Supporting information S3).

*Enzyme linked immunosorbent assay*

To confirm the LC-MS/MS results, enzyme linked immune sorbent assays (ELISA) were performed for *alpha-actinin-4* (ACTN4) and *pyruvate kinase isozyme M1/M2* (PKM2), according to the manufacturer’s instructions. A human ACTN4 ELISA kit (Cusabio Biotech Co. LTD.) and a human PKM2 ELISA kit (USCNK Life Science Inc.) were used to quantify ACTN4 and PKM2 in cervical vaginal washes from healthy (n=16) and HPV-infected (n=12) females (both pre- and post-menopausal). Additionally, several longitudinal samples (n= 29) were also tested for these two candidate biomarkers. According to the manufacturers, the minimal detectable doses of ACTN4 and PKM2 are less than 7.8 pg/ml and 0.144 ng/ml, respectively, and no significant cross-reactivity or interference was observed. All absorption measurements were performed in triplicate at 450 nm using an EnVision 2103 spectrophotometer (PerkinElmer, Zaventem, Belgium).

References

1. Elias JE, Gygi SP. Target-decoy search strategy for increased confidence in large-scale protein identifications by mass spectrometry. *Nat Methods* 2007; **4(3)**:207-214.

2. Zybailov BL, Florens L, Washburn MP. Quantitative shotgun proteomics using a protease with broad specificity and normalized spectral abundance factors. *Mol Biosyst* 2007; **3(5)**:354-360.

3. Liu H, Sadygov RG, Yates JR, III. A model for random sampling and estimation of relative protein abundance in shotgun proteomics. *Anal Chem* 2004; **76(14)**:4193-4201.

4. Zybailov B, Mosley AL, Sardiu ME, Coleman MK, Florens L, Washburn MP. Statistical analysis of membrane proteome expression changes in Saccharomyces cerevisiae. *J Proteome Res* 2006; **5(9)**:2339-2347.

5. Ishihama Y, Oda Y, Tabata T, Sato T, Nagasu T, Rappsilber J*, et al.* Exponentially modified protein abundance index (emPAI) for estimation of absolute protein amount in proteomics by the number of sequenced peptides per protein. *Mol Cell Proteomics* 2005; **4(9)**:1265-1272.

6. Krokhin OV, Craig R, Spicer V, Ens W, Standing KG, Beavis RC*, et al.* An improved model for prediction of retention times of tryptic peptides in ion pair reversed-phase HPLC: its application to protein peptide mapping by off-line HPLC-MALDI MS. *Mol Cell Proteomics* 2004; **3(9)**:908-919.
